# Piercing the veil on the functioning and effectiveness of district health system governance structures: perspectives from a South African province

**DOI:** 10.1186/s12961-023-01044-z

**Published:** 2023-08-31

**Authors:** Khanyisile Tshabalala, Laetitia C. Rispel

**Affiliations:** 1 Department of Public Health Medicine, School of Health Systems and Public Health, Faculty of Health Sciences, University of Pretoria, 31 Bophelo Rd, Prinshof, Pretoria, South Africa; 2 Department of Community Health, School of Public Health, Faculty of Health Sciences, University of the Witwatersrand, Johannesburg, 27 St Andrew’s Road, Parktown, 2193 South Africa; 3 Centre for Health Policy & South African Research Chairs Initiative (SARChI), School of Public Health, Faculty of Health Sciences, University of the Witwatersrand, Johannesburg, 27 St Andrew’s Road, Parktown, 2193 South Africa

**Keywords:** District health system, Governance, Accountability, Universal health coverage, Gauteng

## Abstract

**Background:**

Leadership and governance are critical for achieving universal health coverage (UHC). In South Africa, aspirations for UHC are expressed through the proposed National Health Insurance (NHI) system, which underscores the importance of primary health care, delivered through the district health system (DHS). Consequently, the aim of this study was to determine the existence of legislated District Health Councils (DHCs) in Gauteng Province (GP), and the perceptions of council members on the functioning and effectiveness of these structures.

**Methods:**

This was a mixed-methods, cross-sectional study in GP’s five districts. The population of interest was members of existing governance structures who completed an electronic-self-administered questionnaire (SAQ). Using a seven-point Likert scale, the SAQ focuses on members’ perceptions on the functioning and effectiveness of the governance structures. In-depth interviews with the chairpersons of the DHCs and its technical committees complemented the survey. STATA® 13 and thematic analysis were used to analyze the survey data and interviews respectively.

**Results:**

Only three districts had constituted DHCs. The survey response rate was 73%. The mean score for perceived functioning of the structures was 4.5 (SD = 0.7) and 4.8. (SD = 0.7) for perceived effectiveness. The interviews found that a collaborative district health development approach facilitated governance. In contrast, fraught inter-governmental relations fueled by the complexity of governing across two spheres of government, political differences, and contestations over limited resources constrained DHS governance. Both the survey and interviews identified gaps in accountability to communities.

**Conclusion:**

In light of South Africa’s move toward NHI, strengthening DHS governance is imperative. The governance gaps identified need to be addressed to ensure support for the implementation of UHC reforms.

## Key messages

1. In 2018, only three of the five health districts in the Gauteng Province of South Africa established district health councils, the governance structures for primary health care.
2. The mean score for the perceived functioning of the District Health Council (DHC) was 4.5 out of 7 (SD = 0.7), with the three lowest scoring items being for orientation of DHC members (score 3.8; SD = 2.1), punctuality of meetings (score 4.4 SD = 1.8), and the regular review of DHS performance data (score 4.7 SD = 1.9).
3. The mean score for perceived effectiveness of the DHC was 4.8 out of 7 (SD = 0.7) with the lowest scoring items for tension among members of committees and management (score 3.4 SD = 1.6), the existence of criteria to monitor progress towards goals (score 4.6 SD = 1.7), and accountability to communities (score 4.6 SD = 1.7).
4. The interviews found that a collaborative district health development approach facilitated governance. In contrast, fraught inter-governmental relations fuelled by the complexity of governing across two spheres of government, exacerbated by political differences, and contestations over limited resources constrained DHS governance.
5. Both the survey and interviews identified gaps in accountability to communities.

## Introduction

The Sustainable Development Goals (SDGs) underscore the critical role of governance in achieving social and economic development [1]. The World Health Organization (WHO) defines health system governance as “the existence of strategic policy frameworks, combined with effective oversight, coalition building, regulation, attention to systems design, and accountability” [2]. Brinkerhoff and Bossert expanded the definition of governance to include the interactions, roles, and responsibilities of societal actors both within and outside the health sector [3].

Governance and effective leadership are essential for the optimal functioning of health systems to enable universal health coverage (UHC) and for improved population health outcomes [2]. An ecological study to examine the relationship between governance and under-five mortality rate in 149 countries found that those countries with higher scores on six dimensions of governance had lower under-five mortality rates [4]. Another study focusing on sub-Saharan African countries found that public spending on health improves health outcomes, but the impact of such spending is mediated by the quality of governance [5].

Given the centrality of governance to achieving the SDGs [1], some scholars have proposed frameworks to assess and improve health system governance [6, 7]. Several studies have focused on the types and/or roles of governance structures [8, 9], community participation and accountability, and the challenges of governance [10–13]. Bismark et al. described obstacles to good governance experienced by health service boards in Victoria, Australia. These researchers concluded that these structures were motivated to ensure good governance, but their efforts were tempered by skills gaps of members, as well as the unavailability of tools to monitor their activities [12]. In Brazil, Health Councils have been established at the national, state and municipal levels to provide a platform for citizens to get involved in the monitoring of health policies and health-care delivery [8]. However, a study found that there is poor representation of community members, and follow-up of decisions made in meetings [8]. Masefield and colleagues’ qualitative study of stakeholder perceptions on governance of healthcare in Malawi, identified challenges of accountability, health resource management, unequal power and stakeholder engagement in decision-making [13].

In South Africa, the fundamental principles, structures, and mechanisms of governance are enshrined in the Constitution [14]. The Constitution outlines the roles and responsibilities of national, provincial, and local government, with health services listed as a concurrent functional area of national and provincial levels [14]. The National Health Act underscores the principles of cooperative and sound governance and makes provision for the establishment of the district health system (DHS) [15].

In terms of the Act, the provincial government is responsible for the delivery of PHC services in health districts, the boundaries of which are coterminous with those of local government or municipalities [15]. In practice, both the provincial and local government (especially in metropolitan areas) provide PHC services [16]. Section 31 of the Act provides a framework for the establishment of the governance structures of the DHS, called district health councils (DHCs). In line with the prescripts of the Act, the Member of the Executive Council (MEC) for Health (or provincial health minister) together with the MEC for Local Government have the responsibility for the appointment of DHC members [15].

The DHS is the main vehicle for the delivery of primary health care (PHC), which is the stated foundation of the South African health system [15]. This principle was re-affirmed in the White Paper on the National Health Insurance (NHI) system [17]. The NHI, a health financing system designed to pool funds in both the public and private health sectors, is South Africa’s vehicle for UHC, to ensure equitable, quality health care access, irrespective of socio-economic status [18]. However, the success of the NHI depends on a robust health system, including well-functioning and effective DHS governance structures.

Since democracy in 1994, South Africa’s DHS has been the subject of intense policy and/or scholarly attention [19–28]. DHS progress is illustrated by an enabling policy and legal framework, the establishment of an integrated national public health system, the removal of racial and financial barriers (such as user fees) in access to PHC, the implementation of priority health programmes, and relatively generous public sector funding [29]. However, the vision of a fully functional DHS to ensure the delivery of quality, equitable PHC services has not been realised [28]. Progress has been hampered by a combination of factors, including policy uncertainty, resource constraints, poor leadership, management and governance, and fragmentation of service delivery between provincial and local government health departments [30, 31]. Using the UHC service coverage indicator proposed by Hogan et al., further DHS challenges are illustrated by the 2019 overall UHC service coverage index for South Africa of 58.3, ranging from 53.3 for North West province to 59.2 for Gauteng province [32, 33]. There were also wide variations in the UHC coverage index for the districts, with inequities between urban and rural districts, and in service capacity and health care access [32].

Several studies in South Africa have highlighted the potential of the DHS to improve population health and address the social determinants of health, as well as the potential of community structures such as clinic committees to enhance community participation and accountability [10, 34, 35]. Simultaneously, these studies have revealed the challenges of cooperative governance and decentralisation and the constraints to community participation, including the lack of role clarity, power imbalances between government officials and community members, and the appropriateness of the selection of community members onto these structures [10, 35]. However, there is a dearth of research on whether these DHS governance structures (i.e., the district health councils) exist as envisaged in the National Health Act, and the perceptions of key health policy actors on the functioning and effectiveness of these councils.

Hence, the aim of the study was to assess the existence of district health councils and the perceived functioning and effectiveness of these DHS governance structures in the Gauteng Province of South Africa. The rationale for this study was threefold. Firstly, good governance at the district level is important because of the legislative imperative of a co-operative governance system and management of health services, enshrined in the Constitution [15]. Secondly, the study aimed to generate new knowledge on whether the DHS structures exist according to the National Health Act and the perceptions of those involved in these governance structures in Gauteng Province (GP). In addition, Ford and Ihrke have argued that governance is a human enterprise, and concepts such as functioning, effectiveness and accountability, cannot be measured without considering the perceptions of those involved in governing [36]. Lastly, the study contributes to the growing body of health policy and systems research on DHS governance.

## Methodology

### Conceptual framework

In this study, we applied the WHO’s definition on health systems governance [2] and we used South Africa’s National Health Act as the departure point for our study to assess the existence of DHCs and their perceived functioning and effectiveness in the Gauteng Province of South Africa.

We combined this legal framework (i.e. the National Health Act) with the model of Brinkerhoff and Bossert that focuses on the different policy actors in health systems, the interaction between them, distribution of roles and responsibilities among them and their ability and willingness to fulfil these roles and responsibilities. The Brinkerhoff and Bossert model identifies three categories of health policy actors- politicians and policy makers, the health service providers, and service users [3]. We also drew on the “Perceptions are reality framework” of Ford and Ihrke which states that notions of functioning and effectiveness depend on the perceptions of both the governed and those involved in governing [36].

Although Brinkerhoff and Bossert (2014) and Ford and Ihrke (2019) highlight the role and/or perspectives of service users or those who are governed, this study excludes service users or community members. The reasons were that many other South African studies have focused on the perspectives of clinic committees, and their contribution to district health governance [11, 37, 38]. Additionally, time and budget constraints influenced the scope of the study.

### The study setting

The study was conducted in the GP of South Africa, which comprises the largest share of the South African population, with an estimated 16.1 million people (26.6%) residing in its boundaries [39]. The province is also the centre of the country’s economic development, and contributes approximately one third of South Africa’s Gross Domestic Product (GDP) [40]. Hence, DHS developments and the functioning and effectiveness of governance structures in Gauteng are of strategic importance to the rest of the country.

**Fig. 1:**
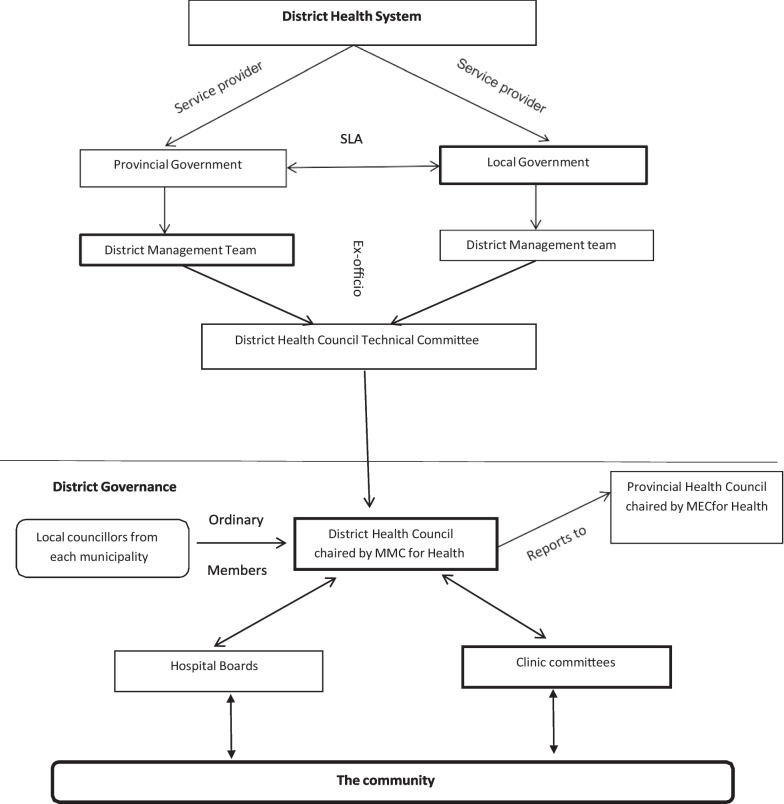
Overview of the DHS; role players and governance structures. Source: Adapted from Health Systems Trust, 2007 [37]. SLA: service level agreement; MMC: Member of the Mayoral Committee; MEC: Member of the Executive Council (provincial minister)

Gauteng is divided into five health districts, three of which are metropolitan municipalities (City of Johannesburg, City of Tshwane and Ekurhuleni) and two are district municipalities (Sedibeng and West Rand). The district municipalities are each further divided into three local municipalities. The de facto organisation of the DHS, the governance structures and key role players are depicted graphically in Fig. 1.

As can be seen from Fig. 1, both the provincial and local government provide PHC services, facilitated by a service level agreement (SLA) between the two spheres of government [15]. In each district, both provincial and local governments are responsible for the management of personal health services. Each district therefore has a provincial manager i.e., a chief director or director, and local government manager i.e. Head of Health at its helm. Both provincial and local government have their respective district management teams (DMTs) to manage district health services. At the district level, District Management Teams (DMTs) are responsible for the planning and management of all PHC services within the districts [15].

### Study design

We conducted a cross-sectional study, using mixed methods: a survey among members of the DHCs and its technical support structure, called the District Health Technical Committees (DHTCTs), and in-depth interviews with the chairpersons of the DHCs and the DHTCTs. For the sake of clarity, we describe the survey first, followed by the qualitative component.

### Perceived functioning and effectiveness survey

#### Study population and sampling

The study population consisted of all the members of the DHCs and members of its technical committee in the five Gauteng health districts (or municipalities) for the period from January until December 2017. The inclusion criteria for survey participants were membership of a DHC or DHCTC in Gauteng and having served at least 3 months as a member of the relevant structure. All the individuals who met the eligibility criteria were invited to participate in the study; hence there was no sampling (*n* = 115).

#### Data collection instrument

Following an extensive literature review, we developed a self-administered questionnaire (SAQ) in English for the survey. The SAQ incorporated relevant elements of the WHO Tool for assessing the operationality of district health systems [41]. The SAQ was divided into three sections: socio-demographics (5 items); perceptions regarding functioning of the governance structures (17 items), and perceptions regarding effectiveness of the governance structures (12 items). Each statement on functioning and effectiveness was scored on a 7-point Likert scale between 1 (strongly disagree) to 7 (strongly agree).

Before implementation, we pre-tested the tool with two members of the DHTCTs. Following testing, adjustments to the wording of two statements were made on the SAQ before the commencement of the study. The responses of those who participated in the pre-test were excluded from the main study.

#### Data collection

During 2018, we conducted the survey using REDCap (Research Electronic Data Capture), a secure, web-based application hosted by the University of the Witwatersrand, Johannesburg for building and managing online surveys and databases [42]. Before the survey, an appointment was made with each participant to enlist participation and facilitate the completion of the survey.

Following informed consent, each participant completed the survey using a hand-held electronic device. In those instances, where we were unable to secure an appointment, we delivered a hard- copy and collected the questionnaire upon completion. As a last option, an electronic link was sent to participants who could not confirm a physical meeting via email for completion.

### Data analysis

Data were imported into STATA® 13 for analysis. We calculated Cronbach’s alpha coefficients on the SAQ to determine reliability and coherence between items. The overall Cronbach alpha score for reliability for the 29 items questionnaire for governance was 0.81. This score indicates good reliability as evidenced by the high inter-item correlation.

We used descriptive statistics to analyse the socio-demographic data. Age being a continuous variable was described using the mean and standard deviation. Categorical variables such as sex, district represented, governance structure type, and portfolio on the governance structure were described using percentages. The duration of membership was described using means and standard deviation. We checked the minutes of meetings held between Jan and Dec 2017 to validate meeting dates reported by participants for the period under review. The content of the minutes was not analysed.

The analysis of participants’ perceptions of the functioning and effectiveness of governance structures was done at different levels. Descriptive statistics (means and standard deviation) were used to describe perceived functioning and perceived effectiveness scores. The items on the questionnaire that had been asked negatively were individually reversed in the analysis to calculate the appropriate means. In STATA, the alpha command was then used to calculate the reliability of the 17-item scale measuring functioning as well as generate the overall mean score for perceived functioning based on the construct. Using the alpha command, a score is created for every observation for which there is a response to at least one item. The summative score is divided by the number of items over which the sum is calculated. Similarly, the alpha command in STATA was again used to calculate the reliability scale of the 12 items measuring effectiveness as well as calculate the overall mean score for perceived effectiveness. The maximum possible score per item was seven and the minimum score one.

A two-sample t-test and ANOVA were used to assess for significant differences in mean perceived functioning scores (from the Likert-scale) between groups categorised according to socio-demographic variables, governance structure type, member type, gender, and district. The same was done for the perceived effectiveness score. The significance level was set at 5%.

We conducted linear regression analyses to assess the association between socio- demographic characteristics and perceived functioning and effectiveness scores, respectively. The two outcome variables were perceived functioning and perceived effectiveness score. Both outcome scores were numerical values. The explanatory variables included in the models were: age (categorised in 10-year intervals), gender, governance structure type, district, portfolio, and whether an individual had orientation on the district health system. These variables were selected based on the study objectives as well as recommendations from previous research assessing factors that influence governance [43]. The R2 value measuring proportion of variance explained was calculated as part of the model. Finally, the *F*-test for the overall model and the coefficient estimates for each predictor were calculated. All tests were conducted at 5% significance levels.

### Qualitative component

#### Population of interest

The purpose of the qualitative component was to explore the perspectives of policy actors or stakeholders on the functioning and effectiveness of the DHS governance structures, including the roles of these structures, factors influencing the functioning or effectiveness of these structures, as well as their challenges and achievements. Given the critical role of the chairperson in steering meetings of the governance structures and ensuring that these are conducted in line with the National Health Act, the population of interest was the chairpersons of the DHC or the DHCTC in the five districts (*n* = 10).

#### Data collection tool

For the qualitative component, we developed a semi-structured interview guide for the in-depth interviews with chairpersons of the DHCs and DHCTCs. The interview guide (8 items) focused the roles of the structure, relationships between or among members of the governance structures, community members, and politicians, achievements as well as challenges.

#### Data collection

We contacted each chairperson of the governance structures to set up a suitable date and time for the interview. Following informed consent, all the interviews were conducted face to face in English, using the interview guide. The questions were open-ended allowing the participants to direct the flow of responses. Consent was also obtained to have the interviews audio recorded. Each interview lasted between 30 and 60 min, depending on the responses of the participants. In addition, detailed field notes were made following each interview.

#### Data analysis

Recordings of the interviews were transcribed verbatim, and transcriptions kept electronically. The audio files and transcripts were stored on a password-protected computer and will be destroyed after the period prescribed by the HREC.

The transcribed data were analysed using thematic analysis. The analysis was an iterative process beginning with familiarization with the data through reading and re-reading three of the transcripts. The narrative text was then coded. Through this process numerous codes were identified. These codes were then grouped and organised into categories which were then defined as themes and sub-themes. Credibility and trustworthiness were established through inter-coder agreement between the researcher, one other researcher, and the two co-authors through the independent coding of a sample of the transcripts. The four individuals then compared the coding and inter-coder agreement was confirmed when consensus on the themes was reached. Once there was agreement on the themes, the remainder of the transcripts were analysed, using a combination of hand-coding and MaxQDA (Verbi software, 2021).

### Data triangulation and integration of quantitative and qualitative findings

We used conceptual, methodological and data triangulation to integrate the quantitative and qualitative components of this study [44]. As described earlier, we combined the prescripts of the NHA, with the theoretical frameworks of Brinkerhoff and Bossert and Ford and Ihrke. Methodological triangulation consisted of the collection and analysis of both quantitative and qualitative data to answer the research objectives. Data triangulation consisted of a comparison of the survey findings with the qualitative findings from the interviews with the chairpersons to reach, followed by integration and interpretation to reach conclusions on the perceived functioning and effectiveness of governance structures.

## Results

### Existence of governance structures

Only three out of the five districts in Gauteng-Sedibeng, West Rand, and Tshwane districts had formally constituted DHCs. The DHCs consist of health officials from both provincial and local government, as well as members of council (politicians at local government level) from local municipalities within the districts. The respective Members of the Mayoral Committee (MMCs) for health in each district chair the DHC.

The District Health Council Technical Committees (DHCTC) which serve as technical advisory committees, were present in all five districts. These technical committees consist of health officials from both provincial and local government. In all the districts, these structures are chaired on a rotational basis by the director or chief director from provincial government, and the head of health from local government. Only three districts—Ekurhuleni, Sedibeng and Tshwane—were willing to share the minutes of the meetings held in the preceding 12 months. These minutes confirmed that meetings had been held every month for the DHCTCs and on a quarterly basis for the DHCs.

### Socio-demographic characteristics

A total of 93 eligible participants participated in the survey (93/115), yielding a response rate of 73%. The mean age of participants in the study was 54 years (SD 7.4). Female respondents constituted 58% of the total sample. Members of the DHTCTs constituted 66% of the total sample while DHC members constituted 34%. The majority of participants were health managers or officials (82%) and the remaining participants were politicians (12%). The mean time served as a member of a governance structure was 6 years (SD ± 4.05).

### Perceptions of functioning of governance structures

The mean score for functioning was 4.5 (SD ± 0.74) out of a possible score of 7. The lowest scoring items on the scale related to members’ punctuality for meetings, whether the committee regularly review data on DHS performance, and whether individuals had received orientation on the district health system. The highest scoring items on the scale were individual understanding on district health services, clear understanding of their role on the structure, and active participation in meetings. The scores are shown in Tables 1 and 2.

**Table 1:** Perception scores on the functioning of DHS governance structures

Item (*N* = 93)	Min	Max	Mean (SD)
Overall perception score for functioning	2.2	5.7	4.5 (0.7)
Schedule of meetings received for the year	1	7	5.6 (1.7)
Agenda for meetings clear	1	7	5.3 (1.5)
Meetings start on time	1	7	4.4 (1.8)
Receive documents for meeting timeously	1	7	4.7 (1.8)
Decisions are taken at every meeting	1	7	5.0 (1.5)
Council/Committee decisions are transparent	1	7	5.4 (1.3)
Council/Committee follows up on recommendations made in previous meetings	1	7	5.0 (1.6)
Clear on role on the council/committee	1	7	5.9 (1.0)
Clear on role of sub-committees	1	7	5.0 (1.3)
Received orientation on DHS	1	7	3.8 (2.1)
Personally understand discussions about DHS	1	7	6.0 (1.0)
Participate actively in meetings	1	7	5.9 (0.9)
Have access to facts that guide decision making at every meeting	1	7	5.1 (1.5)
Regularly review data on DHS performance	1	7	4.7 (1.9)
Health outcomes for the district discussed at council/committee meetings	1	7	5.1 (1.6)
Member perceives hospital boards are important to the DHS	1	7	5.7 (1.1)

**Table 2:** Perception scores effectiveness of DHS governance structures

Item (*N* = 93)	Min	Max	Mean (SD)
Overall perception score for effectiveness	1	5.5	4.8 (0.7)
Chairperson keeps members focused on DHS developments	1	7	5.1 (1.5)
All committed to co-operative governance	1	7	5.0 (1.7)
All participate in development of district health plan	1	7	5.6 (1.4)
There is tension among council/committee members and the provincial executive management	1	7	3.4 (1.6)
There is tension among council/committee members and local government managers	1	7	3.4 (1.6)
Personal knowledge on budget for district health services	1	7	5.3 (1.8)
Council/committee is accountable to community	1	7	4.6(1.7)
Council/committee has a good working relationship with MEC for health	1	7	5.0 (1.2)
Council/committee has criteria to monitor progress toward its goals	1	7	4.6 (1.7)
Collectively examine progress against agreed upon targets	1	7	5.2 (1.5)
Collectively interrogate deviations from targets	1	7	5.0 (1.5)
Collectively interrogate deviations from budget	1	7	5.0 (1.7)

### Perceptions of effectiveness of the governance structures

The mean score for effectiveness was 4.8 (SD ± 0.70) out of a possible 7. The lowest scoring items on the scale related to structures not having criteria to monitor progress toward their goals and accountability to community. The variable on tension among structure members received a score that is mid-point on the 7-point scale. The highest scoring items on the scale were participation in district health planning, knowledge and engagement with district health budget, and engagement on targets for district.

### Predictors of perceived functioning and effectiveness scores

There was a significant difference in perceived functioning scores between structure type (*p* = 0.05) and a marginally significant difference in scores between districts (*p* = 0.08). The smaller district municipalities obtained higher mean scores than the metropolitan municipalities (*p* = 0.08). Similarly, for effectiveness, there was a significant difference in score between the districts (*p* < 0.01) with the smaller district municipalities having a higher mean score than the metropolitan municipalities. The data are shown in Table 3 and 4.

**Table 3:** Predictors of functioning scores

Characteristic	Co-efficient	95% CI	Adjusted *p*-value
Age	0.09	− 0.13 to 0.31	0.45
Gender			
Female	Ref		0.17
Male	0.08	− 0.23 to 0.41	
Governance structure			
DHC	Ref		0.33
DHCTC	− 0.094	− 0.47 to 0.28	
District			
Ekurhuleni	Ref		
Johannesburg	0.49	− 0.01 to 0.98	
Tshwane	0.33	− 0.15 to 0.82	0.05
West Rand	0.70	0.09 to 1.30	
Sedibeng	0.73	0.19 to 1.28	
Portfolio			
Chairperson	Ref		0.30
Politician	− 0.54	− 1.26 to 0.17	
Ex-officio member	− 0.29	− 0.78 to 0.19	
Membership time	0.05	− 0.03 to 0.04	0.78

**Table 4:** Predictors of effectiveness

Characteristic	Co-efficient	95% CI	Adjusted *p*-value
Age	0.11	− 0.08 to 0.32	0.24
Gender			
Male	Ref		0.20
Female	0.19	− 0.11 to 0.48	
Governance structure			
DHC	Ref		0.53
DHCTC	− 0.12	− 0.49 to 0.26	
District			
Ekurhuleni	Ref		
Johannesburg	0.64	0.19–1.18	0.005*
Tshwane	0.18	− 0.26 to 0.63	
West Rand	0.85	0.29 to 1.41	
Sedibeng	0.66	0.12 to 1.10	
Portfolio			
Chairperson	Ref		
Politician	− 0.28	− 0.93 to 0.37	0.61
Ex-officio member	− 0.03	− 0.47 to 0.42	
Membership time	− 0.01	− 0.04 to 0.03	0.79

### Qualitative results

Although inter-related and not mutually exclusive, the major themes that emerged from the interviews were: collaborative district health development; fragile governance arrangements and functionality; fraught intergovernmental relationships; resource constraints and contestations; and peripheral community participation or accountability. For the sake of clarity, each theme is highlighted separately.

### Collaborative district health development

This theme captures the achievements in DHS enunciated by participants. Participants felt that having a shared vision for the DHS and goals for PHC resulted in achievements for the DHS, and by proxy its governance structures. The complexity of dual authority providing single service was acknowledged by many, but the commitment to provide services to people came out strongly as a motivator for cooperation.

“*We need each other, we need cooperation in order to maximize our coverage of the area and to serve all our people as…as far as possible*” (key informant 2)

“*There’s a clear change that we want to bring in people’s lives. So, we need to have a plan and a vision. That’s why we have to meet because that vision we need then to share it with the politicians but also give the politicians something they can bite on. So, we have to meet and agree, what we are all going to be selling to the politicians for us to be able to move and get somewhere.*” (Key informant 5)

### Fragile governance arrangements and functionality

This theme highlights the governance arrangements found at the time of the study. Only three DHC were established formally, but with difficulties even in the other two. Participants articulated their frustrations with the delays in setting up the structures or committees, the duplication of management structures (local versus provincial government), and the practice of working in silos. Participants pointed to the fact that this is driven by different priorities between provincial and local government, as well as different political priorities. According to participants, differences are difficult to bridge when they are along political lines and alliances.

*Unfortunately, we did not start off on a very good footing. We have not had a single sitting since the new administration. You know the psychological climate between us and Province was not good, you know, especially at a political level.”* (Key informant 6).

The fragilities went beyond political tensions, and included issues of discontentment with the process of provincialisation (the taking over of all health facilities in a district by provincial government from local government), decision-making, as well as lack of synergies in infrastructure planning and procurement processes between local government and provincial government.

*“Before provincialisation it was fine because they still had uh…control over health, but since health was taken from them… They just felt… It…it…it must do hands off. It’s not their baby. And before, funds were allocated to them, that is why they were cooperating because they were getting money from us”* (Key Informant 7)

### Fraught intergovernmental relationships (IGR)

This theme, linked to the fragility of the structures, explores the working relationships that were further complicated by different power dynamics, egos and attitudes of individuals, which create a tense working environment. Participants reported that they had to navigate and negotiate personality differences and communication styles before delving into issues affecting district health services. Despite this, the chairpersons appeared to have developed mechanisms in dealing with the differences in order to put service delivery at the fore.

*“Sometimes our egos control how we respond to issues in a meeting because sometimes we don’t feel like…we don’t want to be seen to be taking instructions from others in front of our subordinates. Then you engage separately in an informal way. Maybe you can have lunch or breakfast together just to talk outside the work pressures.”* (Key informant 1)

*“Like any working relationship I normally say it’s…it’s a marriage where you are…you are in a marriage, sometimes you want to walk away, but you’ve got children to look after so you can’t walk away.”* (Key informant 5)

### Peripheral community participation or accountability

Participants highlighted several gaps in accountability particularly to communities, such as insufficient community awareness of governance structures, insufficient linkages between district health system governance and community structures, and with surrounding communities. Community participation was largely described as a marginal activity, mostly restricted to dealing with complaints, and engaging informally through facility visits.

*“There’s no interaction whatsoever [referring to the community]. I wonder how many residents are aware of this entity called the District Health Council. Only the few informed or that might have been involved with it. We haven’t even contemplated what is our responsibility, or what should we communicate with, or how should we involve them…I think, if I can say what I sense, is that we need not or we do not want to or we do not have to communicate to give feedback in any way. We should take into account their interest, the health needs and as executing authorities, province, ourselves…we need to do what’s best for them.”* (Key informant 2)

Several participants also highlighted that there were other platforms for direct community engagement which they participated in. This included the Gauteng Provincial Government Ntirhisano (working together) initiative which gives residents an opportunity to engage with politicians and officials at ward level on matters of concern to them. Similarly, all municipalities produce an Integrated Development Plan (IDP) that in principle considers community concerns and integrates them into the plans for the municipality. While several participants praised these initiatives as a positive, some raised concern over the perceived disjuncture between issues raised at community level, issues discussed in the District Health Councils, and issues addressed in the integrated plans.

*“A governance related issue…its lack of integration of the IDP related issues, especially community issues into the District Health Council as the key Council itself. We appear to be working vertically. And there’s no synergy between issues that were raised at the community level that we found and District Health Council for admission to the Provincial Health Council.”* (Key informant 9)

### Resource constraints and contestations

This theme enunciates the identified resource constraints for DHS including but not limited to lack of finances, lack of staffing, and equipment. Beyond the constraints, contestations in terms of resource allocation and unfunded mandates were explored. The majority of participants identified insufficient health resources as a barrier for effectiveness. Participants lamented the fact that despite the high burden of disease and constant need to expand services, the resources required to ensure responsiveness often lagged behind. In addition, participants identified introduction of new priority programs and political initiatives without the accompanying resources.

*“We’ve got political imperatives and targets that are set, and you don’t have funding for them. National at the moment has given the policy on universal test and treat* [of HIV infection]. *It means everybody that walks in there that gets tested. But as a municipality, did anybody look whether we’ve got the capacity in terms of person power and the space in our clinics?”* (Key Informant 5)

## Discussion

This is one of the first studies that examined the establishment of DHS governance structures in accordance with South Africa’s National Health Act, and the perceptions of the members on the functioning and effectiveness of these governance structures. We combined the frameworks of Ford and Ihrke [36] that emphasizes the value of perceptions of those governing and that of Brinkerhoff and Bossert [3] that highlights the different societal actors in health systems, the interaction between them, and the impact on governance.

The study found that only three of the five Gauteng districts had established DHCs. The two large cities of Johannesburg and Ekurhuleni did not have formal DHCs. Reasons cited for the lack of these structures included political tensions and difficult inter-personal relationships. These findings are similar to those of a comparative study conducted in Kenya and Indonesia in 2018, which found that political differences and power dynamics constrained the ability of people to work together and threatened good governance [45]. We found that the smaller district municipalities obtained higher mean scores for both perceived functioning and perceived effectiveness, compared to the metropolitan municipalities, two of which did not have established structures. Consequently, the absence of the DHCs in the two cities of Johannesburg and Ekurhuleni is of major concern. Furthermore, both these large cities are of strategic importance to health system developments in Gauteng specifically, and South Africa in general.

Our study illustrates that the existence of enabling legislation is insufficient to foster optimal functioning. This was also found in an exploratory study on the adequacy of health councils in Brazil, showing that these councils did not meet the minimum conditions necessary to fulfil their role [46]. Given that there is an association between the quality of governance and health outcomes [5], the absence of these structures in two large metropolitan areas of the Gauteng province is likely to hamper efforts to improve PHC service delivery and the health outcomes of communities served in these two health districts.

From the survey results, the mean functioning score for the existing governance structures for all districts was 4.5 out of a possible 7, suggesting room for improvement. Nonetheless, the members’ self-reported understanding of their role in governance, collaborative district health development and commitment to service delivery emphasized in the qualitative interviews are encouraging. Both the quantitative and qualitative components reported high participation in district health planning, which was highlighted as a facilitator for district health achievements. District managers in South Africa have highlighted the benefits of joint planning as it helps them to overcome fragmentation and encourage teamwork [47].

The mean score for effectiveness was 4.8 out of a possible 7, demonstrating again room for improvement. In the survey, the responses to the items on tensions within the structures were non-committal, as the mean scores were mid-way on the Likert scale (3.4). However, in the interviews, participants highlighted tensions along political lines, spheres of government, as well as at an interpersonal level, thus contributing to the fractured state of governance. The qualitative interviews demonstrate how contestation over resources, budgets, and mandates further strain both functionality and effectiveness. The impact of power relations at the district level on accountability and governance was also found in a 2020 study in Tajikistan, with complex practices of power and contestation over resources within the bureaucracy shaping policy implementation [48].

In the survey results, accountability to community obtained a low score. This was also highlighted in the in-depth interviews as a largely marginal activity. Low accountability to communities is exacerbated by the uncertainty among some participants on how they should interact with communities and what should be communicated to communities. Some participants held the view that they could decide unilaterally what was in the best interest of residents in terms of the delivery of health services. This authoritarian view is not uncommon among political leaders and health officials [3], and has also been found in the 2020 Tajikistan study [48]. Similarly, a 2006 review of community participation in health- care across East and Southern Africa found gaps in community participation and accountability, and the importance of two-way communication [47].

Our study limitations are non-response bias and social desirability bias. Although we took steps to minimize non-response, including extensive consultation with relevant stakeholders, careful explanation of the study to the possible participants, relationship building, and attending relevant governance structures meetings, the overall response rate was 73%. This was due to the fact that two of the five districts did not have formal DHCs and refusal by some study participants. Social desirability bias was minimised by the self-administered questionnaire during the survey, and securing individual appointments for the interviews.

However, our study has numerous strengths. Firstly, methodological strength is the use of mixed methods enabled us to measure perceived scores on functioning and effectiveness and obtain in-depth insights from chairpersons on these aspects. Secondly, our study adds to the discourse on health system governance. It highlights the intersection of fraught inter-governmental relations fuelled by the complexity of governing across two spheres of government, exacerbated by political differences, and contestations over limited resources and DHS governance.

Based on the study findings, we propose a set of recommendations to strengthen DHS governance. South Africa’s National Health Act is enabling in prescribing the structures, roles, and functions of the DHS governance structure. However, the study has demonstrated areas where there is insufficient clarity on how district-level governance structures should interact with each other and with communities. In June 2023, South Africa’s National Parliament approved the NHI Bill, which paves the way for UHC, with its central focus on PHC delivered through the DHS [49]. The Bill does not stipulate the structures and mechanisms for DHS governance, suggesting uncertainty or a policy vacuum on the role of DHCs in the new era [50]. Nonetheless, the reforms envisaged with the implementation of the NHI system present a window of opportunity to revitalise the concept of the DHS system. The DHS in turn has the potential to prioritise the implementation of the PHC approach that underscores community participation, intersectoral collaboration, and health service delivery closest to communities [34]. Hence, we propose the development of national guidelines for DHS governance structures. The purpose of these guidelines would be to clarify the appropriate structures for DHS governance, composition of the structures and what governance structures ought to do, how they differ from district management teams, how the different structures at the district level should interact among themselves, and how these structures should interact with communities to advance functionality and effectiveness. These guidelines could also be used to train the various health policy actors on the principles of good governance. While it is accepted that the development of legislation and guidelines enables good governance, the existence of strategic policy frameworks in the absence of implementation is fruitless. Regardless of what new legislation or policies for governance might come to be, these need to be followed by support from national and provincial government to ensure that they are implemented at the local level. The MEC for health and local government, and MMCs for health in each district should ensure that governance structures are constituted and supported in their functions. Furthermore, it is recommended that the NDoH mandate all provinces to dedicate a line item in the health budget on DHS governance, thus facilitating it’s strengthening and capacity development and members.

## Conclusion

This was one of the first studies to examine the functioning and perceived effectiveness of district health governance structures in Gauteng. The study findings should inform future DHS developments and strengthen DHS governance to support the implementation of the impending UHC reforms in this province. The study also contributes to the discourse on health system governance in South Africa, and in other low-and-middle-income countries. In light of South Africa’s proposed NHI system that seeks to achieve UHC, the strengthening of DHS governance is imperative.

## Data Availability

The datasets used and/or analysed during the current study are available from the corresponding author on reasonable request.

## Abbreviations

DHC: District health council
DHCTC: District Health Council Technical Committee
DHS: District health system
MEC: Member of Executive Council
NHA: National Health Act
PHC: Primary health care
UHC: Universal health coverage
SDGs: Sustainable Development Goals
